# Ray of dawn: Anti-PD-1 immunotherapy enhances the chimeric antigen receptor T-cell therapy in Lymphoma patients

**DOI:** 10.1186/s12885-023-11536-4

**Published:** 2023-10-23

**Authors:** Yuxin Zhou, Wenjing Mu, Chen Wang, Zipeng Zhuo, Yu Xin, Hongxu Li, Changsong Wang

**Affiliations:** 1 Department of Critical Care Medicine, the First Affiliated Hospital of Harbin Medical University, Harbin, Heilongjiang Province 150001 China; 2 Department of Anesthesiology, Zhujiang Hospital of Southern Medical University, Guangdong, Guangzhou Province 510280 China; 3 Department of Critical Care Medicine, the Cancer Hospital of Harbin Medical University, Harbin, Heilongjiang Province 150081 China; 4 Department of Digestive medicine center, The Seventh Affiliated Hospital, Sun Yat-sen University, Shenzhen, 518017 China

**Keywords:** Chimeric antigen receptor T cell therapy, Programmed cell death protein-1, Lymphoma, Efficacy, Safety, Immunotherapy

## Abstract

**Background:**

Chimeric antigen receptor T (CAR-T) cell therapy, a new adoptive cell therapy, has been widely used to treat lymphoma patients. Immune checkpoint blockade may improve the cytotoxicity of CAR-T cells by reducing the failure of CAR-T cells and improving antitumor activity. It has shown promising efficacy.

**Method:**

We searched PubMed, the Cochrane Library, Embase and Web of Science from January 2012 to August 2022 to find data reporting the results of CAR-T cells therapy combined with PD-1 in tumor patients. An updated search was conducted in October 2023. The partial response rate (PR), complete response rate (CR), objective response rate (ORR), mortality rate, and incidence of adverse reactions were calculated.

**Results:**

We analyzed 57 lymphoma patients from 5 clinical trials. The pooled partial, complete and overall response rates were 21% (95% CI 0.06–0.39, I^2^ = 0.37%), 27% (95% CI 0.03–0.60, I^2^ = 60.43%) and 65% (95% CI 0.23–0.98, I^2^ = 76.31%), respectively. The pooled incidence of cytokine release syndrome, neutropenia, fever, and fatigue was estimated to be 57% (95% CI 0.08–0.99, I^2^ = 85.20%), 47% (95% CI 0.14–0.81, I^2^ = 74.17%), 59% (95% CI 0.27–0.89, I^2^ = 60.23%), and 50% (95% CI 0.13–0.87, I^2^ = 73.89%), respectively.

**Conclusion:**

CAR-T-cell therapy combined with anti-PD-1 immunotherapy in the treatment of lymphoma patients has efficacy, and the most common adverse effect is fever.

**Registration:**

The protocol was registered in prospero, with the registration number CRD42022342647.

**Supplementary Information:**

The online version contains supplementary material available at 10.1186/s12885-023-11536-4.

## Introduction

Hematologic malignancies include all types of leukemia, multiple myeloma, and malignant lymphoma, including Hodgkin’s lymphoma (HL) and non-Hodgkin’s lymphoma (NHL). The incidence rate of severe disease is 13.9% in patients diagnosed as hematological malignancy within one year [1, 2], and the prognosis is unfavorable [3]. With the application of molecular targeted drugs and immunotherapy, the prognosis of many patients with hematological malignancies has improved [4, 5]. Chimeric antigen receptor-T (CAR-T) cell therapy, as a new popular method in cancer treatment, has been approved by the US Food and Drug Administration for the treatment of some hematologic malignancies, such as Kymriah (tisagenlecleucel) [6], Yescarta (axicabtagene ciloleucel) [7], Tecartus (brexucabtagene autoleucel) [8], and lisocabtagene maraleucel (liso cel) [9]. It has achieved good clinical therapeutic effects [10].

CAR-T cells are genetically engineered T cells. They are composed of the extracellular antigen binding domain of scFv and transmembrane domain and intracellular signal transduction or costimulatory domain (usually CD3ξ) [11, 12], which trigger a cell activation signal when it encounters a target antigen [13]. Single variable domains on a heavy chain (VHH) are called nanobodies and have now been applied to targeted domains in CARs [14]. However, due to off-target effects, antigen escape, inadequate T-cell proliferation and persistence enhance the inhibitory tumor microenvironment [15, 16]. According to many international and clinical studies, some patients still do not respond to CAR-T therapy [17–19] and experience serious side effects (such as cytokine release syndrome (CRS) and neurotoxicity) [20, 21]. Improving the efficacy of CAR-T cells, reducing the occurrence of side effects, and improving the long-term treatment rate of disease are currently the most concerning issues of CAR-T therapy at present.

Early failure of CAR-T cells is one of the main factors limiting the antitumor efficacy of CAR-expressing T cells. The expression of multiple coinhibitory receptors on T cells may be related to the failure of CAR-T cells [22]. Studies have shown that the expression of programmed cell death protein-1 (PD-1) is upregulated on CAR-T cells [23, 24]. It may reduce the advantage of 4-1BB costimulated CD8 + CAR-T cells in central memory accumulation and long-term antitumor effects [25]. Targeting multiple inhibitory pathways may enhance the efficacy of CAR-T cells [22]. PD-1 is a cell surface receptor that inhibits T-cell inflammatory activity by binding to ligands [25]. Tumor-induced downregulation of T-cell function can be reversed by immune checkpoint inhibitors [26]. It has been reported that PD-1 destruction by genome editing can enhance the antitumor activity of CAR-T cells [27–29] and can also save CAR-T cells from exhaustion and senescence [30, 31]. The overall survival of patients with CAR-T cells was prolonged [32]. CAR-T cells combined with PD-1 have been widely used in preclinical models and clinical trials to enhance the efficacy of cancer therapy. PD-1 blockade is regarded as a promising new idea to improve the function of CAR-T cells. However, the literature still lacks sufficient experimental evidence, and the combined application of the two new immunotherapies is still controversial due to the adverse expansion and short-term clinical remission of T cells and other reasons [33, 34]. Many clinical trials have been conducted to verify the feasibility of CAR-T cells combined with PD-1 in the treatment of lymphoma patients. Our objective was to evaluate the efficacy and safety of this treatment through our systematic review.

## Method

### Literature search strategy

We searched PubMed, the Cochrane Library, Embase and Web of Science from January 2012 to August 2022 to find data reporting the results of CAR-T cells therapy combined with PD-1 in tumor patients. An updated search was conducted in October 2023. The method of combining subject and free words was used for the literature search. The search terms included “Chimeric Antigen Receptor Therapy”, “Immune Checkpoint Inhibitors” and “CAR T-Cell Therapies”. Detailed search strategies are provided in Supplement 1.

### Literature screening and data extraction

The included literature met the following criteria: (1) tumor patients; (2) the experimental group received CAR-T cells combined with PD-1; (3) clinical research; and (4) relevant outcome indicators: partial response rate (PR); complete response rate (CR); objective response rate (ORR); mortality rate; and incidence of adverse reactions. Repeated publications of articles and reviews, conference reports, case reports and animal experiments were excluded.

According to the inclusion and exclusion criteria, two independent researchers selected studies. The third person chose the differences. The extracted literature information included basic information (author, publication year), trial design type, sample size, intervention measures, outcome indicators, etc.

### Statistical analysis

We used Stata 16.0 software for the meta-analysis. The effect size and 95% confidence interval were synthesized by software. *p* < 0.05 was considered statistically significant. The obtained effect values were analyzed for heterogeneity. If there was no significant statistical heterogeneity among studies (*p* > 0.1, *p* < 50%), the fixed effects model was adopted; if statistical heterogeneity existed (*p* < 0.1, *p* ≥ 50%), the random effects model was used to combine the effect size. Only descriptive analysis was performed if the heterogeneity was too obvious and the source could not be determined. Sensitivity analysis was used to determine the stability of each study. All included studies were evaluated using the Joana Brigg’s Institute (JBI) quality assessment scale [35]. (Supplement 2) The methodological quality of each report was evaluated by ranking the risk of bias as high, medium and low. A score of more than 6 out of 9 items was considered low risk.

## Results

**Fig. 1:**
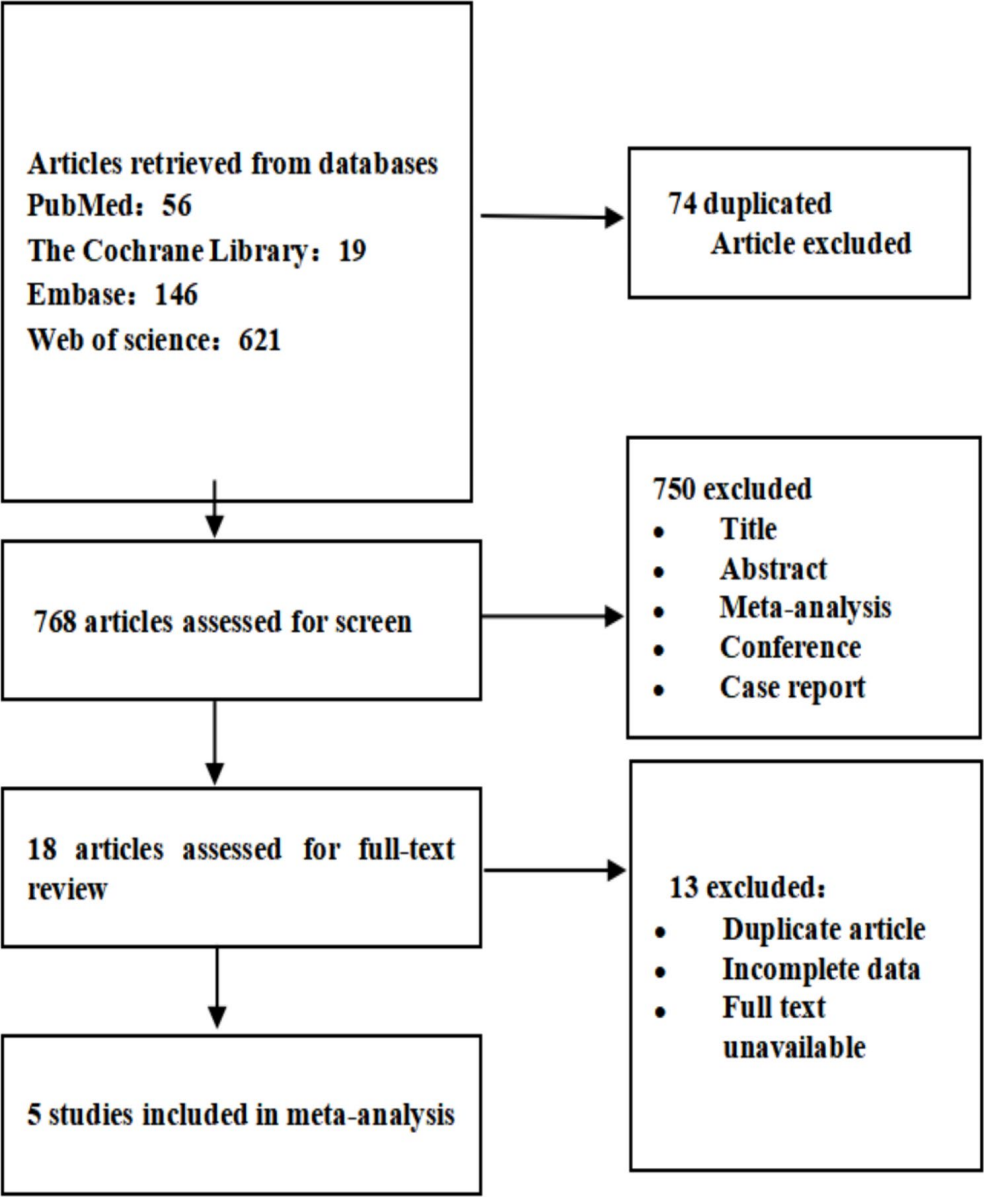
Flow chart of the study selection process

We finally included 5 randomized controlled trials [2, 31, 36–38], which included a total of 57 patients with lymphoma. Twenty patients were excluded due to the absence of combined PD-1 treatment, and 37 patients were eventually included. All patients had adult lymphoma pretreated with low-dose chemotherapy (patient characteristics are shown in Table 1). The indications of the patients in the included studies are as follows: patients with progressive B-cell non-Hodgkin’s lymphoma after anti-CD19 CAR-T-cell infusion [31]; patients with CD19-positive B-cell lymphoma who have progressed or relapsed after chemotherapy [2]; relapsed or refractory diffuse large B-cell lymphoma [36]; relapsed/refractory B-cell lymphoma after CAR-T-cell therapy [36]; and CD30-positive relapsed/refractory patients with lymphoma [38]. Five patients were treated with single-dose autologous anti-CD30 CAR-T cells intravenously, and 32 were treated with 4-1BB costimulated anti-CD19 CAR-T cells. Twenty-one patients were given pembrolizumab, and 11 patients were given nivolumab. The screening process is shown in Fig. 1.

**Table 1:** Basic characteristics of included studies

								Efficacy					Safety			
Study	Median age, year	Study population	Prior therapy lines	PD-1	CAR-T target antigen	costimulatory molecules	PD1 dose	mortality	PFS	PR, n(%)	CR, n(%)	ORR, n (%)	CRS grade ≥ 3, n(%)	CRS grade<3, n(%)	Fever,n(%)	Neutropenia, n(%)
Elise A. Chong.2017	56.7	8	5(3–7)	Pembrolizumab	CD19	41BB	200 mg/3weeks	0	2.2month(0.4–3.2)	1(12%)	1(12%)	2(25%)	1(12.5%)	N	3(38%)	3(38%)
Yaqing Cao.2019	65	11	NA	Nivolumab	CD19	41BB	3 mg/kg	5(45%)	6month(1–14)	4(36%)	5(45%)	9(82%)	N	9(82%)	9(82%)	3(27%)
Xinfeng Chen.2020	59	1	6	Pembrolizumab	CD19	41BB	100 mg/3weeks	0	NR	1(100%)	NR	NR	N	1(100%)	1(100%)	1(100%)
Elise A. Chong.2022	58	12	4(3–8)	Pembrolizumab	CD19	41BB	200 mg/3weeks	0	2.8month(0.4–35.2)	2(21%)	1(8%)	3(25%)	N	1(8.3%)	3(25%)	3(25%)
Sang Wei. 2022	25	5	NA	PD1	CD30	NA	NR	1(20%)	21.5month(3–50)	1(20%)	4(80%)	5(100%)	1(20%)	4(80%)	4(80%)	5(100%)

Abbreviation: progression-freesurvival (PFS); partial response(PR); complete response(CR); overall response rate (ORR); cytokine release syndrome (CRS); none reported(NR); none(N)

**Fig. 2:**
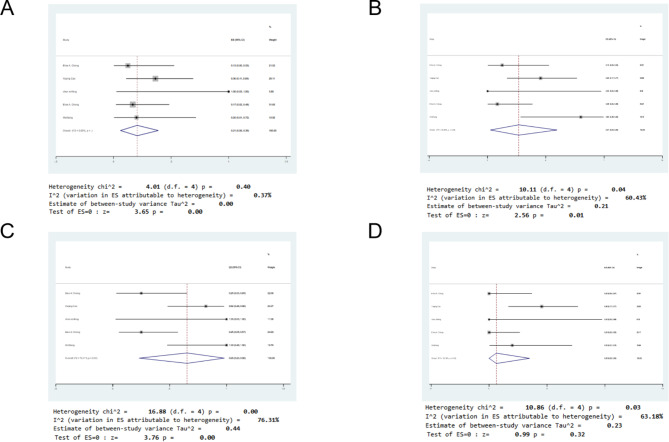
Forest plot of partial response (PR), complete response (CR), objective response rate (ORR), and mortality rate for patients. (**A**) PR, (**B**) CR, (**C**) ORR, (**D**) mortality rate

PR rate: Five studies reported PR, including 37 patients. The heterogeneity analysis results were as follows: *p* = 0.40, I^2^ = 0.37%, indicating low heterogeneity. Therefore, a fixed-effect model was used. The results of the meta-analysis showed that the effect size (95% CI = 0.21 [0.06, 0.39], z = 3.65, *p* < 0.05) was statistically significant. This result suggested that the PR rate of CAR-T cells combined with PD-1 in tumor patients was 21% (Fig. 2A).

CR rate: CR rates were reported in all 5 studies. Thirty-seven patients were included, and 11 patients experienced a complete response. The results of the heterogeneity analysis were as follows: *p* = 0.04, I^2^ = 60.43%, indicating high heterogeneity. Therefore, the random effects model was adopted. The results of the meta-analysis showed that the effect size (95% CI = 0.27 [0.03, 0.60], z = 2.56, *p* < 0.05) was statistically significant. This result suggested that the CR rate of CAR-T cells combined with PD-1 in tumor patients was 27% (Fig. 2B). The CR of 32 patients treated only with anti-CD19 CAR-T cells was 16% [95% CI 0.02,0.35, I^2^ = 30.04%]. (Supplement 3)

The overall ORR was reported in 5 studies, including 37 patients and 20 patients with objective response. The results of the heterogeneity analysis were as follows: *p* < 0.1, I^2^ = 76.31%, indicating high heterogeneity. Therefore, the random effects model was adopted. The results of the meta-analysis showed that the effect size (95% CI = 0.65 [0.23, 0.98], z = 3.76, *p* < 0.05) was statistically significant, suggesting that the objective response rate of CAR-T cells combined with PD-1 therapy in tumor patients was 65% (Fig. 2C). Among them, 6 patients died. The mortality rate of tumor patients treated with CAR-T cells combined with PD-1 was 6% [95% CI 0.00, 0.35], z = 0.99, *p* = 0.32 (Fig. 2D).

### Adverse reactions

**Fig. 3:**
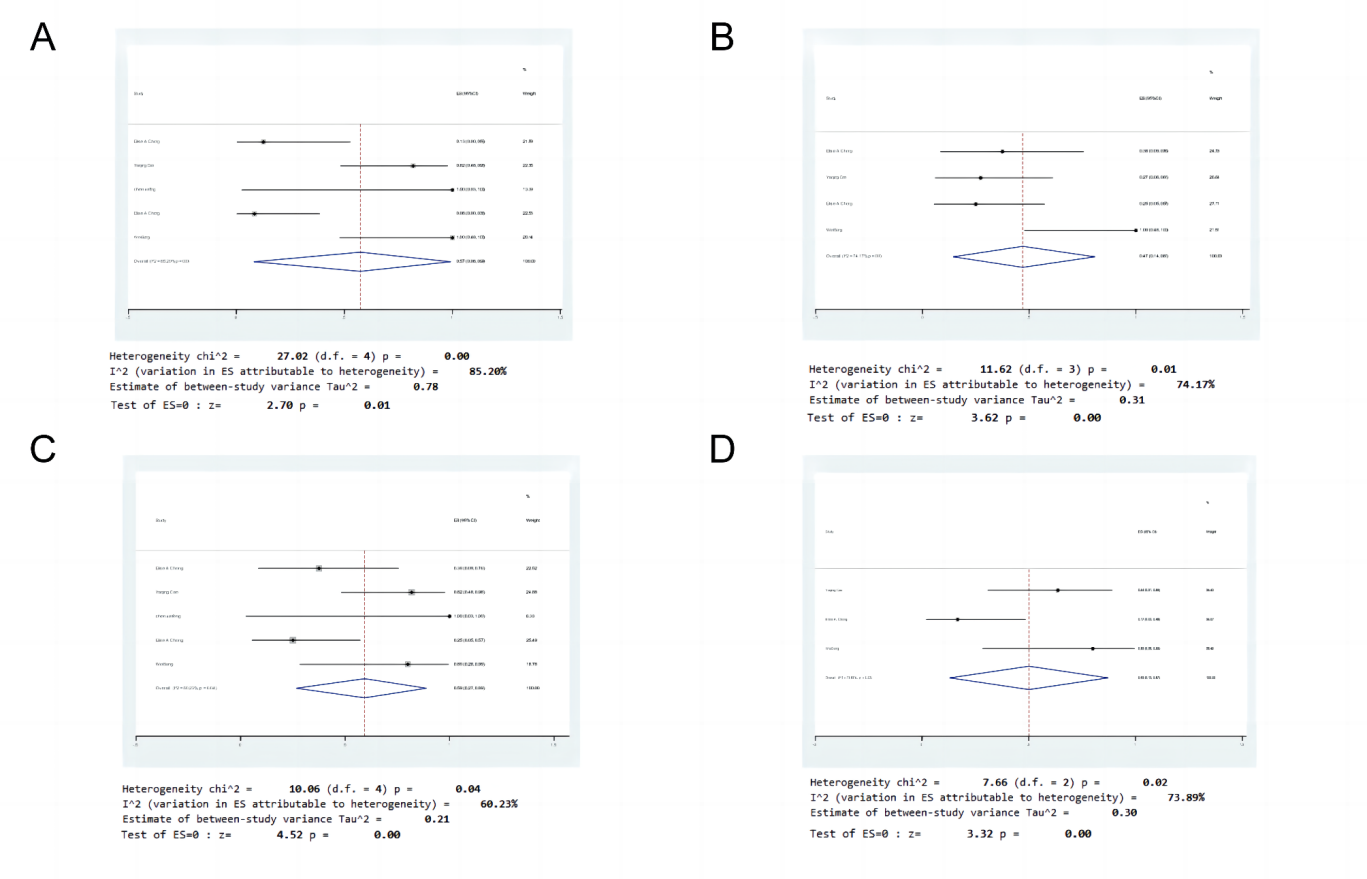
Forest plot of adverse reactions for patients. (**A**) Cytokine release syndrome (CRS), (**B**) neutropenia, (**C**) fever, (**D**) fatigue

CRS is a common side effect of CAR-T therapy. CRS of any grade was reported in 5 studies, and 17 patients were enrolled. The results of the heterogeneity analysis were as follows: *p* < 0.1, I^2^ = 85.20%, indicating high heterogeneity. Therefore, we adopted the random effects model. The results of the meta-analysis showed that the effect size (95% CI = 0.57[0.08, 0.99], z = 2.70, *p* = 0.01) was statistically significant. It was suggested that the incidence of CRS of any grade in tumor patients treated with CAR-T cells combined with PD-1 was 57% (Fig. 3A). Fifteen patients developed grade 1–2 CRS. The results of the heterogeneity analysis were as follows: *p* < 0.1, I2 = 85.23%, indicating high heterogeneity, so the random effects model was adopted. The results of the meta-analysis showed that the effect size (95% CI = 0.43 [0.01, 0.92], z = 2.17, *p* = 0.03) was statistically significant (Supplement 4). These results suggest that the incidence of grade 1–2 CRS in tumor patients treated with CAR-T cells combined with PD-1 was 43%. Only two patients reported grade 3–4 CRS.

Many reports have confirmed that neutropenia is the most common grade 3/4 adverse event. Our study found that 4 studies reported complications of grade 3–4 neutropenia, which occurred in 14 patients (47%) of the 36 patients included. The results of the heterogeneity analysis were *p* = 0.01 and I^2^ = 74.17%, indicating high heterogeneity. Therefore, the random effects model was adopted, and the results of the meta-analysis showed that the effect size (95% CI = 0.47 [0.14, 0.81], z = 3.62, *p* < 0.01) was statistically significant (Fig. 3B).

Other adverse effects were also found. A total of 20 (59% [95% CI 0.27, 0.89, I^2^ = 60.23%] *p* < 0.01) of the 37 patients were found to have a fever (Fig. 3C). Of the 28 patients, 13 (50% [95% CI 0.13, 0.87, I^2^ = 73.89%] *p* < 0.01) had fatigue symptoms (Fig. 3D).

**Fig. 4:**
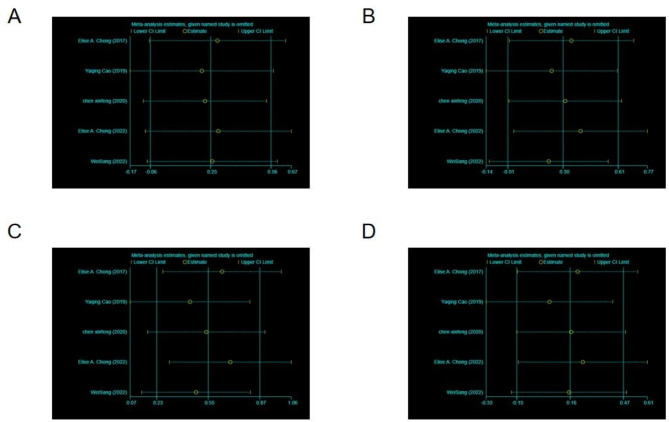
Sensitivity analysis of partial response (PR), complete response (CR), objective response rate (ORR), and mortality rate for patients. (**A**) PR, (**B**) CR, (**C**) ORR, (**D**) mortality rate

**Fig. 5:**
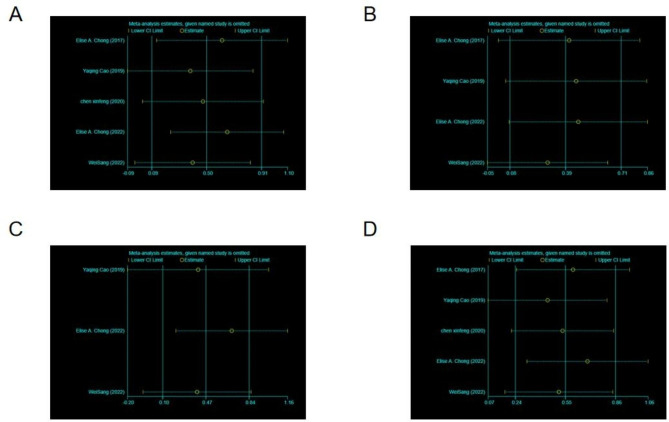
Sensitivity analysis of adverse reactions for patients. (**A**) Cytokine release syndrome (CRS), (** B**) neutropenia, (**C**) fever, (**D**) fatigue

Sensitivity analysis and the evaluation of the bias were performed to eliminate studies using a one-by-one method. We found that the results of the research were not significantly different after excluding and removing the results one by one (Figs. 4 and 5). This finding suggests that the meta-analysis result is stable and reliable. The risk of bias was assessed as low risk in all studies.

## Discussion

Our meta-analysis showed that CAR-T cells combined with PD-1 showed good safety and efficacy in patients with hematologic tumors. The ORR was 65% [95% CI 0.23, 0.98], and the CR was 27% [95% CI 0.03, 0.60]. The CR of anti-CD19 CAR-T cells constructed with 4-1BB costimulation was 16% [95% CI 0.02, 0.35, I^2^ = 30.04%], which was lower than that of all CAR-T-cell treatments. This may be due to the better efficacy of CD30 CAR-T therapy in the treatment of relapsed or refractory HL [39]. However, due to the long cycle of CAR-T therapy, current clinical trials are mostly used in patients with relatively stable hematologic tumors, so the true efficacy of all patients using such therapy may be lower than our results. A meta-analysis revealed that CAR-T therapy resulted in a 46% CR and 66% ORR for B-cell non-Hodgkin lymphoma [40], which is a better clinical outcome than our study. Currently, combination therapy is mainly applied to patients with clinical relapse and no response to single CAR-T therapy or other immunotherapies. Their disease is more complex, and the formation of a tumor immunosuppressive microenvironment affects the amplification of CAR-T cells [37], so the CR is lower than that in other studies on single CAR-T-cell therapy for hematologic tumors. However, studies have shown that the combination of PD-1 inhibitors in patients who do not respond to CAR-T therapy can improve the ORR of some patients [2].

The most common acute toxicity of CAR-T cells is CRS [41]. The incidence of CRS of any grade was 57% (95% CI 0.08, 0.99), and the rating was mainly based on the PENN scale [42]. Class 1–2 CRS accounted for 43% (95% CI 0.01, 0.92). Most grade 1–2 adverse events can be managed with drugs, and the incidence of severe autoimmune events is low. Nonsteroidal anti-inflammatory drugs [43] and corticosteroids can relieve symptoms and control CRS to a certain extent. However, there may be a risk of damage to CAR-T cells [44, 45]. No significant difference was found between anti-CD30 and anti-CD19 outcomes using different surface targets.

Continuous tumor attack can lead to the high expression of PD-1 on the surface of T cells. This leads to the exhaustion of CAR-T cells and the weakening of antitumor effects [46]. By adopting anti-PD-1 CAR-T cells in NSG mice, mouse CAR-T cells showed enhanced antitumor reactivity, and the amplification effect of CAR-T cells was enhanced [32], promoting tumor regression and reducing the incidence of adverse reactions [47, 48]. GUO et al. found that the destruction of endogenous PD-1 in CAR-T cells could enhance the toxicity of T cells, the production of the cytokines IFN-γ and IL-2, and the antitumor effect and survival time of CAR-T cells [49]. The PD-1 pathway may also affect the efficacy of CAR-T cell immunotherapy by preventing CAR-T cells from entering the tumor area [50]. With pembrolizumab after CAR-T cell treatment, all responding patients experienced more than one CAR-T cell amplification peak, and the CAR-T cells lasted longer than nonresponding patients, with only one peak [2]. One case report also showed that PD-1 inhibition could effectively increase diffuse large B-cell lymphoma that did not respond to CAR-T therapy and enhance the expansion of CD19 CAR-T cells [31]. Therefore, it is reasonable to think that PD-1 inhibitors may partially prevent the depletion and functional deterioration of anti-CD19 CAR-T cells.

This systematic review and meta-analysis provides some reference value for clinical treatment. To our knowledge, this is the first study to systematically analyze the efficacy of CAR-T cell therapy combined with PD-1 in the treatment of lymphoma patients. This meta-analysis has some limitations as well. First, the heterogeneous nature of the included studies (paucity of the available literature, dosing differences, lines of prior therapy differences, etc.) might introduce bias. Second, due to the small number of included studies, the sample size was not large enough. Nevertheless, this meta-analysis will be useful to design and prioritize future clinical trials for CAR-T-cell therapy combined with anti-PD-1 immunotherapy.

### Electronic supplementary material

Below is the link to the electronic supplementary material.

Additional File 1: Supplement 1. Retrieval strategy.

Additional File 2: Supplement 2. JBI critical appraisal quality assessment of the case series study.

Additional File 3: Supplement 3. (A). Sensitivity analysis of complete response (CR) for patients with anti-CD19 CAR-T therapy. (B) Forest plot of complete response (CR) for patients with anti-CD19 CAR-T therapy.

Additional File 4: Supplement 4. (A). Sensitivity analysis of 1–2 grade CRS for patients. (B) Forest plot of 1–2 grades CRS for patients.

## Data Availability

The datasets used and/or analysed during the current study available from the corresponding author on reasonable request.
