# Supplementary material for: Ray of dawn: Anti-PD-1 immunotherapy enhances the chimeric antigen receptor T-cell therapy in Lymphoma patients
Source: BMC Cancer. 2023 Oct 23;23:1019. doi: 10.1186/s12885-023-11536-4 (PMC10591343; doi:10.1186/s12885-023-11536-4)
Supplement: Supplementary file 1 — Additional File 1: Supplement 1. Retrieval strategy. [file 12885_2023_11536_MOESM1_ESM.docx]

#### **Retrieval strategy**

| ****Database**** | **Pubmed** |
| --- | --- |
| ****Website**** | **https://pubmed.ncbi.nlm.nih.gov** |
| ****Time**** | **database building - 2022.08** |
| ****Results**** | **56** |
| ****Search details**** | **((("Immunotherapy, Adoptive"[Mesh]) OR ((((((((Chimeric Antigen Receptor Therapy[Title/Abstract]) OR (CAR T-Cell Therapy[Title/Abstract])) OR (CAR T Cell Therapy[Title/Abstract])) OR (CAR T-Cell Therapies[Title/Abstract])) OR (T-Cell Therapies, CAR[Title/Abstract])) OR (T-Cell Therapy, CAR[Title/Abstract])) OR (Therapies, CAR T-Cell[Title/Abstract])) OR (Therapy, CAR T-Cell[Title/Abstract]))) AND (("Immune Checkpoint Inhibitors"[Mesh]) OR ((((((((Checkpoint Inhibitors, Immune[Title/Abstract]) OR (Immune Checkpoint Inhibitor[Title/Abstract])) OR (Immune Checkpoint Blockers[Title/Abstract])) OR (Immune Checkpoint Blockade[Title/Abstract])) OR (Immune Checkpoint Inhibition[Title/Abstract])) OR (PD L1 Inhibitors[Title/Abstract])) OR (PD 1 Inhibitors[Title/Abstract])) OR (CTLA 4 Inhibitors[Title/Abstract])))) AND ((clinical[Title/Abstract] AND trial[Title/Abstract]) OR clinical trials as topic[MeSH Terms] OR clinical trial[Publication Type])** |
| ****Database**** | **Embase** |
| ****Website**** | **https://www.embase.com** |
| ****Time**** | **database building - 2022.08** |
| ****Results**** | **146** |
| ****Search details**** | No. Query  #10. #3 AND #6 AND #9  #9. #7 OR #8  #8. 'clinical trial':ti,ab,kw OR 'clinical trial':ti,ab,kw  #7. 'clinical trial (topic)'/exp  #6. #4 OR #5  #5. 'immune checkpoint inhibitor'/exp  #4. 'checkpoint inhibitors, immune':ti,ab,kw OR 'immune checkpoint inhibitor':ti,ab,kw OR 'immune checkpoint blockers':ti,ab,kw OR 'immune checkpoint blockade':ti,ab,kw OR 'immune checkpoint inhibition':ti,ab,kw OR 'pd 1 inhibitors':ti,ab,kw OR 'pd 1 inhibitors':ti,ab,kw OR 'ctla 4 inhibitors':ti,ab,kw  #3. #1 OR #2  #2. 'adoptive immunotherapy'/exp  #1. 'chimeric antigen receptor therapy':ti,ab,kw OR 'car t-cell therapy':ti,ab,kw OR 'car t cell therapy':ti,ab,kw OR 'car t-cell therapies':ti,ab,kw OR 't-cell therapies, car':ti,ab,kw OR 't-cell therapy, car':ti,ab,kw OR 'therapies, car t-cell':ti,ab,kw OR 'therapy, car t-cell':ti,ab,kw |
| ****Database**** | Web of science |
| ****Website**** | **http://www.webofscience.com** |
| ****Time**** | **database building - 2022.08** |
| ****Results**** | 621 |
| ****Search details**** | #1:Chimeric Antigen Receptor Therapy(Topic) OR CAR T-cell Therapy(Topic) OR CART cell Therapy(Topic) OR CAR T cell Therapy(Topic) OR T-cell Therapy CAR(Topic) 16926  #2:Checkpoint Inhibitors, Immune(Topic) OR Immune Checkpoint Inhibitor(Topic) Immune Checkpoint Blockers(Topic) OR **PD L1 Inhibitors**(Topic) **OR PD 1 Inhibitors**(Topic) **OR CTLA 4 Inhibitors**(Topic) 67597  #3:clinical trial(Topic) OR clinical trial (Publication/Source Titles) 1535691  #4: #1 AND #2 AND #3 621 |
| ****Database**** | Cochrane |
| ****Website**** | https://www.cochrane.org |
| ****Time**** | **database building - 2022.08** |
| ****Results**** | 19 |
| ****Search details**** | ID Search Hits  #1 MeSH descriptor: [Immunotherapy, Adoptive] explode all trees 122  #2 (Chimeric Antigen Receptor Therapy):ti,ab,kw OR (CAR T-Cell Therapy):ti,ab,kw OR (CAR T Cell Therapy):ti,ab,kw OR (CAR T-Cell Therapies):ti,ab,kw OR (T-Cell Therapies, CAR):ti,ab,kw (Word variations have been searched) 278  #3 (T-Cell Therapy, CAR):ti,ab,kw OR (Therapies, CAR T-Cell):ti,ab,kw OR (Therapy, CAR T-Cell):ti,ab,kw (Word variations have been searched) 184  #4 MeSH descriptor: [Immune Checkpoint Inhibitors] explode all trees 75  #5 (Checkpoint Inhibitors, Immune):ti,ab,kw OR (Immune Checkpoint Inhibitor):ti,ab,kw OR (Immune Checkpoint Blockers):ti,ab,kw OR (Immune Checkpoint Blockade):ti,ab,kw OR (Immune Checkpoint Inhibition):ti,ab,kw (Word variations have been searched) 1721  #6 (PD L1 Inhibitors):ti,ab,kw OR (PD 1 Inhibitors):ti,ab,kw OR (CTLA 4 Inhibitors):ti,ab,kw (Word variations have been searched) 5677  #7 #1 or #2 or #3 391  #8 #4 or #5 or #6 6591  #9 MeSH descriptor: [Clinical Trials as Topic] explode all trees 48676  #10 (clinical):ti,ab,kw AND (trial):ti,ab,kw (Word variations have been searched) 713234  #11 (clinical trial):pt (Word variations have been searched) 333718  #12 #9 or #10 or #11 943531  #13 #7 and #8 and #12 19 |
