# Supplementary material for: Ray of dawn: Anti-PD-1 immunotherapy enhances the chimeric antigen receptor T-cell therapy in Lymphoma patients
Source: BMC Cancer. 2023 Oct 23;23:1019. doi: 10.1186/s12885-023-11536-4 (PMC10591343; doi:10.1186/s12885-023-11536-4)
Supplement: Supplementary file 2 — Additional File 2: Supplement 2. JBI critical appraisal quality assessment of the case series study. [file 12885_2023_11536_MOESM2_ESM.docx]

Supplement 2: JBI critical appraisal quality assessment of the case series study.

| Author, year of publication | Q1 | Q2 | Q3 | Q4 | Q5 | Q6 | Q7 | Q8 | Q9 | Total score |
| --- | --- | --- | --- | --- | --- | --- | --- | --- | --- | --- |
| Elise A. Chong, 2017 | Y | Y | Y | NA | Y | NR | Y | Y | Y | 7 |
| Yaqing Cao, 2019 | Y | Y | Y | Y | Y | Y | Y | Y | Y | 9 |
| chen xinfeng. 2020 | Y | Y | Y | Y | Y | NR | Y | Y | Y | 8 |
| Elise A. Chong, 2022 | Y | Y | Y | Y | Y | Y | NA | Y | Y | 8 |
| Wei Sang, 2022 | Y | Y | NA | Y | NR | Y | Y | NA | Y | 6 |

Key: Y = Yes; NR = Not reported, NA = Not appropriate

**Question codes:**

1. Was the sample frame appropriate to address the target population?

2. Were study participants sampled in an appropriate way?

3. Was the sample size adequate?

4. Were the study subjects and the setting described in detail?

5. Was the data analysis conducted with sufficient coverage of the identified sample?

6. Were valid methods used for the identification of the condition?

7. Was the condition measured in a standard, reliable way for all participants?

8. Was there appropriate statistical analysis?

9. Was the response rate adequate, and if not, was the low response rate managed appropriately?
