# Supplementary material for: Ray of dawn: Anti-PD-1 immunotherapy enhances the chimeric antigen receptor T-cell therapy in Lymphoma patients
Source: BMC Cancer. 2023 Oct 23;23:1019. doi: 10.1186/s12885-023-11536-4 (PMC10591343; doi:10.1186/s12885-023-11536-4)
Supplement: Supplementary file 3 — Additional File 3: Supplement 3. (A). Sensitivity analysis of complete response (CR) for patients with anti-CD19 CAR-T therapy. (B) Forest plot of complete response (CR) for patients with anti-CD19 CAR-T therapy. [file 12885_2023_11536_MOESM3_ESM.pdf]

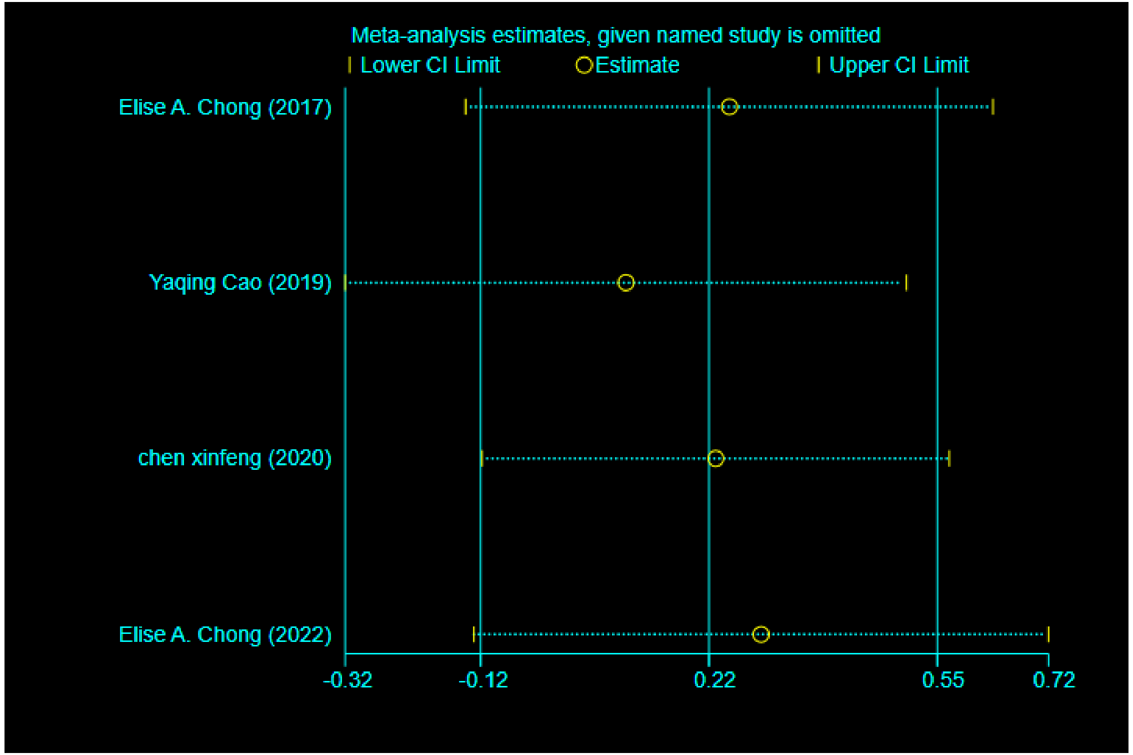

A

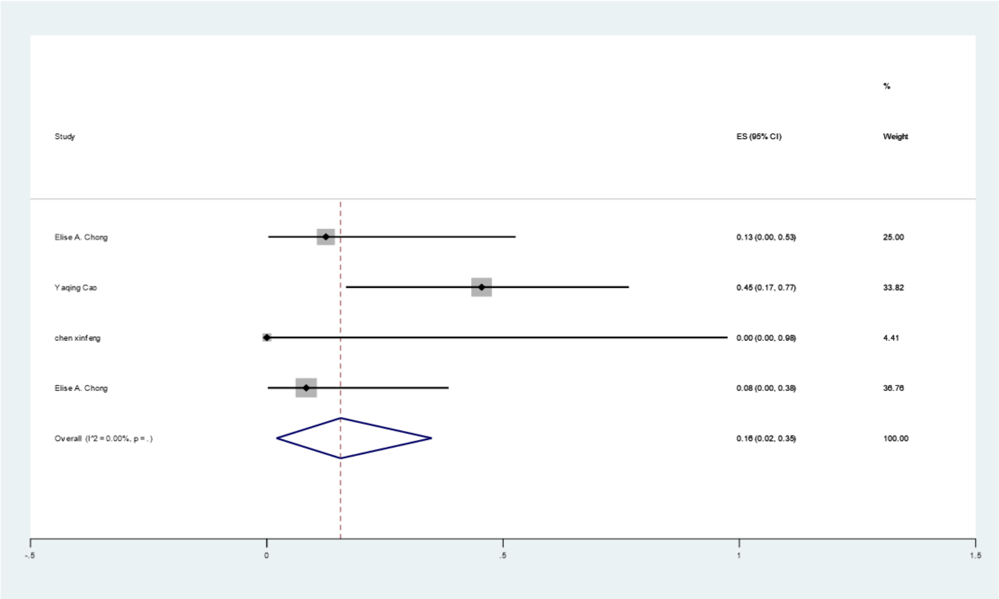


```
Heterogeneity chi^2 =       4.29 (d.f. = 3) p =       0.23
I^2 (variation in ES attributable to heterogeneity) =     30.04%
Estimate of between-study variance Tau^2 =       0.06
Test of ES=0 : z=      2.79 p =       0.01
```

B
