# Supplementary material for: Ray of dawn: Anti-PD-1 immunotherapy enhances the chimeric antigen receptor T-cell therapy in Lymphoma patients
Source: BMC Cancer. 2023 Oct 23;23:1019. doi: 10.1186/s12885-023-11536-4 (PMC10591343; doi:10.1186/s12885-023-11536-4)
Supplement: Supplementary file 4 — Additional File 4: Supplement 4. (A). Sensitivity analysis of 1–2 grade CRS for patients. (B) Forest plot of 1–2 grades CRS for patients. [file 12885_2023_11536_MOESM4_ESM.pdf]

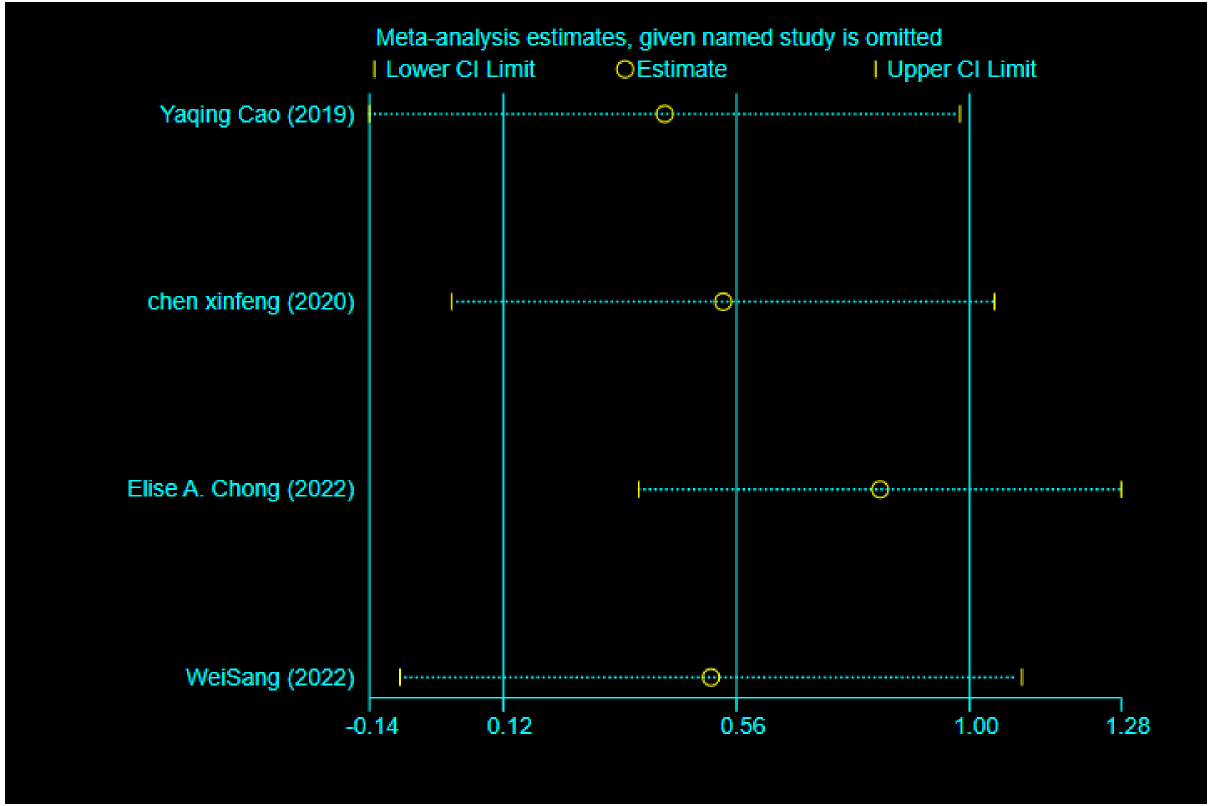

A

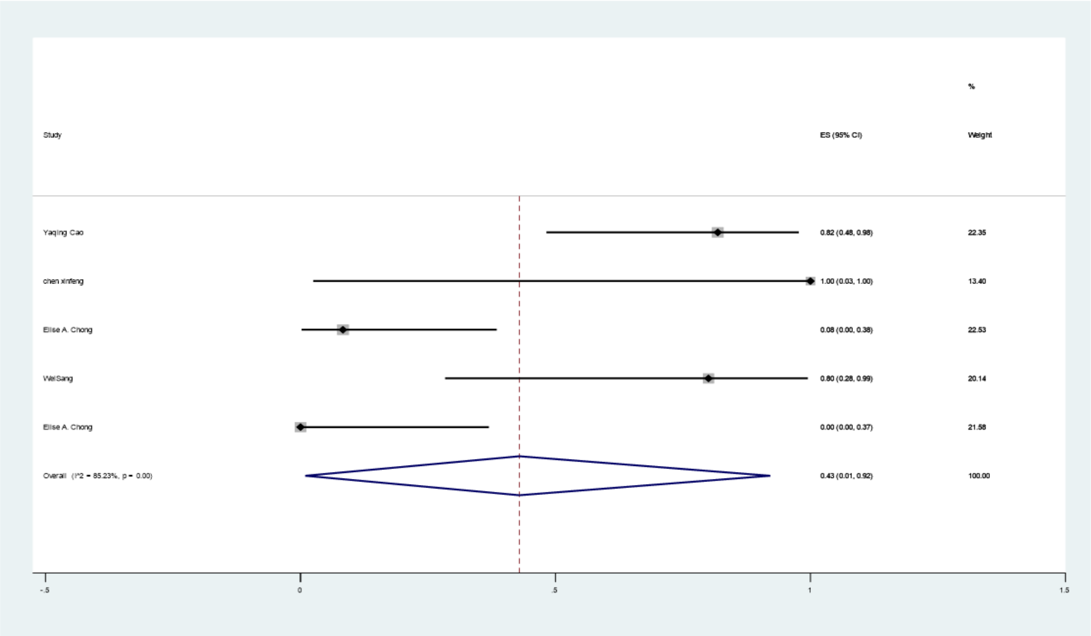


```
Heterogeneity chi^2 =      27.08 (d.f. = 4) p =       0.00
I^2 (variation in ES attributable to heterogeneity) =     85.23%
Estimate of between-study variance Tau^2 =       0.78
Test of ES=0 : z=       2.17 p =       0.03
```

B
